# Association of P450 Oxidoreductase Gene Polymorphism with Tacrolimus Pharmacokinetics in Renal Transplant Recipients: A Systematic Review and Meta-Analysis

**DOI:** 10.3390/pharmaceutics14020261

**Published:** 2022-01-22

**Authors:** Da-Hoon Lee, Hana Lee, Ha-Young Yoon, Jeong Yee, Hye-Sun Gwak

**Affiliations:** 1 College of Pharmacy and Graduate School of Pharmaceutical Sciences, Ewha Womans University, Seoul 03760, Korea; hhooonnn@ewhain.net (D.-H.L.); hayoungdymphnayoon@gmail.com (H.-Y.Y.); 2 Graduate School of Clinical Biohealth, Ewha Womans University, Seoul 03760, Korea; lhn9095@naver.com

**Keywords:** tacrolimus, POR, pharmacokinetics, kidney transplant

## Abstract

There are conflicting results regarding the effect of the P450 oxidoreductase (*POR*) *28 genotype on the tacrolimus (TAC) pharmacokinetics (PKs) during the early post-transplantation period in adult renal transplant recipients. Thus, we characterized the impact of POR*28 on TAC PKs. We conducted a systematic review on the association between POR*28 and PKs of TAC in adult renal transplant recipients. Structured searches were conducted using PubMed, Web of Science, and Embase. TAC standardized trough concentration (ng/mL per mg/kg) data were extracted. Mean differences (MD) and their corresponding 95% confidence intervals (CIs) were used to identify the differences between the POR*28 genotype and PKs of TAC. The subgroup analysis was conducted according to *CYP3A5* expression status. Six studies (n = 1061) were included. TAC standardized trough concentrations were significantly lower in recipients with the POR*28 allele compared to recipients with POR*1/*1 (MD: 8.30 ng/mL per mg/kg; 95% CI: 1.93, 14.67; *p* = 0.01). In the subgroup analysis, TAC standardized trough concentrations were lower for subjects who were POR*28 carriers than those who were POR*1/*1 in *CYP3A5* expressers (MD: 20.21 ng/mL per mg/kg; 95% CI: 16.85, 23.56; *p* < 0.00001). No significant difference between POR*28 carriers and POR*1/*1 was found in the *CYP3A5* non-expressers. The results of our meta-analysis demonstrated a definite correlation between the POR*28 genotype and PKs of TAC. Patients carrying the POR*28 allele may require a higher dose of TAC to achieve target levels compared to those with POR*1/*1, especially in *CYP3A5* expressers.

## 1. Introduction

Tacrolimus (TAC), one of the calcineurin inhibitors, is commonly used as an immunosuppressant to prevent acute organ rejection after kidney transplantation [1]. TAC has a narrow therapeutic index and wide interindividual pharmacokinetic (PK) variability. Thus, TAC administration requires therapeutic drug monitoring (TDM) to enhance efficacy and to avoid side effects [1,2,3,4]. Although TDM is widely practiced, some patients are exposed to sub- or supra-therapeutic concentrations of TAC, thereby increasing their risk of acute organ rejection or toxicity within a week after transplantation [5].

TAC is metabolized by cytochrome P450 (CYP), especially CYP3A5 [6]. CYP3A5*3 (c.219-237A>G; rs776746) is a critical predictor of CYP3A5 activity [7,8], and several studies reported that *CYP3A5* non-expressers (CYP3A5*3/*3) are related to decreased metabolizing functions and higher TAC trough concentrations compared with *CYP3A5* expressers (CYP3A5*1/*1 or CYP3A5*1/*3) [9,10,11,12].

Recently, further attention has been given to P450 oxidoreductase (POR), which transfers electrons from nicotinamide-adenine-dinucleotide phosphate-oxidase to CYP enzymes, inducing CYP expression and affecting TAC metabolism [13,14]. Among several single nucleotide polymorphisms (SNPs) of *POR,* the most common variant is POR*28 (c.1508 C>T, rs1057868). According to an in vitro study, this SNP was associated with increased CYP activity, including CYP1A2, CYP2C19, CYP3A4, and CYP3A5 [15]. Previous studies have investigated the role of POR*28 in the PKs of TAC and reported that patients carrying POR*28 exhibited lower trough concentrations of TAC and required higher TAC doses than wild-type patients (POR*1/*1) [16,17,18]. However, the results of previous studies are conflicting due to their small sample sizes. Therefore, we conducted a systematic review and meta-analysis of the existing studies to determine the effects of POR*28 on TAC trough concentrations in renal transplant patients.

## 2. Materials and Methods

### 2.1. Search Strategy and Study Selection

This study was performed according to the Preferred Reporting Items for Systematic Reviews and Meta-Analyses (PRISMA) guidelines [19]. We performed a comprehensive search of three electronic databases (PubMed, Web of Science, and Embase) on 16 July 2021 using the following search terms: (tacrolimus OR FK506 OR FK-506 OR (calcineurin inhibitor) OR Prograf OR immunosuppress*) AND ((kidney transplant*) OR (kidney graft*) OR (kidney allograft*) OR (renal transplant*) OR (renal graft*) OR (renal allograft*)) AND (POR OR (p450 oxidoreductase) OR (cytochrome p450 oxidoreductase) OR CYPOR) AND (polymorph* OR variant* OR mutation* OR genotyp* OR phenotyp* OR haplotyp* OR SNP OR rs1057868 OR Ala503Val OR A503V) (Table 1).

Studies were selected if (1) the studies focused on the effects of the POR*28 genotype on renal transplant patients receiving TAC; (2) the studies had TAC PK data expressed as standardized trough concentration (ng/mL per mg/kg); and (3) the articles were published in English. Standardized trough concentration was determined as the concentration adjusted by the dose per body weight. Studies were excluded if they were (1) abstracts, reviews, editorials, or letters; (2) in vitro or in vivo studies; (3) studies performed on pediatric patients; or (4) studies from which we were unable to extract outcome data.

After removing duplicate studies, two authors independently excluded irrelevant studies by reviewing the titles and abstracts. Then, full-text articles were assessed for inclusion. Any inconsistencies were resolved by consensus between the two authors.

### 2.2. Data Extraction and Study Quality Assessment

Two reviewers independently extracted data using a preconceived data extraction spreadsheet. The following information was included: name of the first author, publication year, ethnicity, patient numbers, percentage of males, mean age, mean body weight, follow-up day, TAC initial dose, target trough level, concomitant drugs, and method of genotyping and quantification. Two reviewers assessed the study’s quality using the Newcastle–Ottawa scale (NOS) tool [20]. The NOS tool is based on three domains: the selection of exposed and unexposed subjects (0–4 points), comparability of study groups (0–2 points), and outcome assessment (0–3 points). In terms of comparability, if *CYP3A5* expression and age were adjusted for the analysis, we rated them with 1 point for each.

### 2.3. Statistical Analysis

Mean differences (MD) and their corresponding 95% confidence intervals (CIs) were used to identify the differences between the POR*28 genotype and PKs of TAC, and the Z-test was performed to detect the statistically significant differences between two groups. To calculate pooled estimates, we extracted the mean and standard deviation. If the studies only reported the median and interquartile range, the formulas by Wan et al. [21] were used to estimate the mean and standard deviation. Data presented as log-transformed mean and standard deviation were converted to the raw scale using the methodology of Higgins et al. [22].

We assessed the heterogeneity across studies using the chi-square test and I^2^ statistics [23], and I^2^ > 50% was regarded as indicating significant heterogeneity. The fixed-effect model was used if there was no significant heterogeneity; otherwise, the random-effects model was used. When we confirmed heterogeneity, a sensitivity analysis was conducted by omitting each study in turn to assess the influence of individual studies. To detect publication bias, Begg’s rank correlation test and Egger’s regression test were performed using R Studio software (version 3.6.0; R Foundation for Statistical Computing, Vienna, Austria) [24,25]. As the effects of POR*28 can depend on the expression status of *CYP3A5*, a subgroup analysis was conducted according to *CYP3A5* expression status. The meta-analysis was performed using Review Manager 5.4 (The Cochrane Collaboration, Copenhagen, Denmark). Statistical significance was defined as a *p*-value < 0.05.

## 3. Results

**Figure 1:**
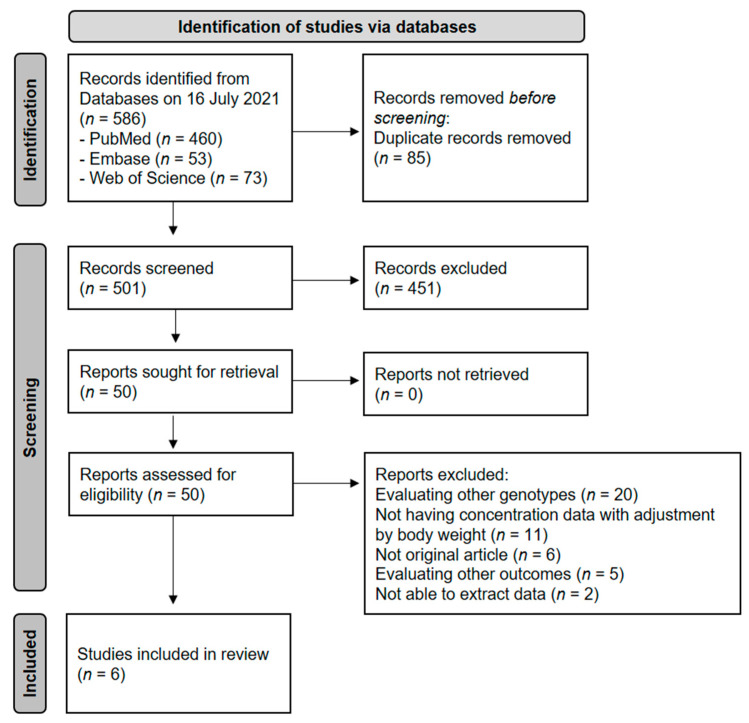
A flow diagram of study selection.

Our initial search yielded 586 studies, 501 of which remained after duplicates were removed. After excluding 451 articles based on their titles and abstracts, we assessed the full text of 50 studies. Among them, 44 studies were excluded for the following reasons: evaluating other genotypes (*n* = 20), not having concentration data with adjustment by body weight (*n* = 11), not an original article (*n* = 6), evaluating other outcomes (*n* = 5), and not able to extract data (*n* = 2). Finally, six studies [26,27,28,29,30,31] involving 1061 patients were included in the meta-analysis (Figure 1). The characteristics of these studies are summarized in Table 2. The studies were published between 2014 and 2018, and all were cohort studies. Four of the six studies were conducted with Asian populations, one with Caucasians, and the other with multiethnic groups. The mean age of the patients was 43.3 years (range 40.0–49.5). Quality scores evaluated using the NOS ranged from 7 to 9.

**Figure 2:**
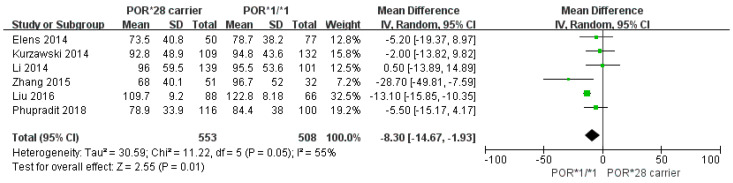
A forest plot showing the association between POR*28 carriers and standardized trough concentration (ng/mL per mg/kg) of tacrolimus.

**Figure 3:**
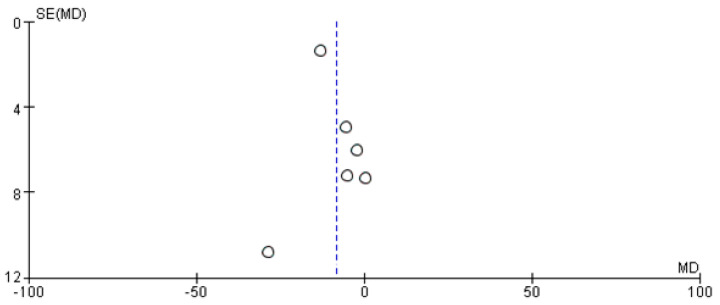
A funnel plot showing the association between POR*28 carriers and standardized trough concentration (ng/mL per mg/kg) of tacrolimus. SE: standard error, MD: mean difference.

The results of a meta-analysis investigating POR*28 and standardized trough concentrations of TAC are shown in Figure 2. POR*28 carriers showed a 8.30 ng/mL per mg/kg lower concentration of TAC when compared with POR*1/*1 carriers (95% CI: 1.93, 14.67; *p* = 0.01; I^2^ = 55%). The funnel plot was asymmetrical (Figure 3), and Begg’s test and Egger’s test indicated no evidence of publication bias (*p* = 0.573 and *p* = 0.293, respectively). In the sensitivity analysis, the exclusion of Liu et al. led to a loss of statistical significance (Table 3).

**Figure 4:**
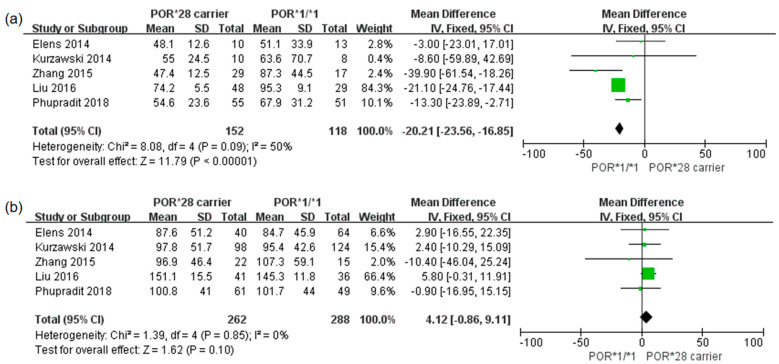
Forest plots with *CYP3A5* expressing and *CYP3A5* non-expressing subgroups showing the association between POR*28 carriers and standardized trough concentration (ng/mL per mg/kg) of tacrolimus. (**a**) *CYP3A5* expressers; (**b**) *CYP3A5* non-expressers.

Five studies reported the influence of the POR*28 genotype on the standardized trough concentrations of TAC according to *CYP3A5* expression status [26,27,29,30,31]. There were 270 *CYP3A5* expressers (CYP3A5*1/*1 or *1/*3) and 550 *CYP3A5* non-expressers (CYP3A5*3/*3). In the *CYP3A5* expressing subgroup, the TAC standardized trough concentration was 20.21 ng/mL per mg/kg lower for POR*28 carriers than for POR*1/*1 carriers (95% CI: 16.85, 23.56; *p* < 0.00001; I^2^ = 50%; Figure 4a). However, in the *CYP3A5* non-expressing subgroup, POR*28 was not associated with the TAC standardized trough concentration (MD: 4.12 ng/mL per mg/kg, 95% CI: −9.11, 0.86; *p* = 0.1; I^2^ = 0%; Figure 4b).

## 4. Discussion

This is the first meta-analysis investigating the association between the POR*28 polymorphism and the standardized initial trough concentration of TAC in adult renal transplant recipients. The results showed that POR*28 carriers had a lower standardized trough concentration of TAC when compared with POR*1/*1 carriers. This association was increased in *CYP3A5* expressers; however, POR*28 did not affect the TAC concentration in *CYP3A5* non-expressers.

POR*28, a missense variant of *POR*, is the most common variant observed in about 28% of all alleles [32]. This variant is present in the flavin adenine dinucleotide (FAD) binding site, thereby affecting POR and CYP interactions [33]. In vitro studies demonstrated that POR*28 affects CYP3A4 activity in a substrate-specific manner [34,35]. Several PK studies demonstrated that POR*28 is related to increased CYP3A activity. The study of Oneda et al. [36], which investigated CYP3A in vivo activity using midazolam, showed that POR*28/*28 was related to a 1.6-fold increase in hepatic CYP3A activity. Yang et al. [37] also showed that POR*28 was associated with increased hepatic CYP3A activity. In line with previous findings, our results regarding increased CYP3A activity might be explained by the effects of POR*28.

Several studies have reported that decreased exposure to TAC within a week after transplantation was associated with acute organ rejection. Kuypers et al. [38] reported that patients with an area under the concentration curve of 0–12 h (AUC_(0–12)_) below 200 ng·h/mL had a higher risk of acute rejection when compared with those with a higher AUC_(0–12)_. Borobia et al. [39] also showed that patients with acute organ rejection had lower TAC trough concentrations than those without acute organ rejection. Our meta-analysis demonstrating that patients carrying the POR*28 allele had decreased TAC concentrations indicates POR*28 is an important factor in predicting acute organ rejection.

According to the subgroup analysis in this meta-analysis, POR*28 effects on the TAC concentration varied by *CYP3A5* expression status, which is consistent with previous studies. For example, according to Jonge et al. [18], *CYP3A5* expressers carrying the POR*28 allele required an approximately 25% higher TAC dose than *CYP3A5* expressers with POR*1/*1, although the POR*28 allele did not affect TAC doses in *CYP3A5* non-expressers. Gijsen et al. [40] reported that, in *CYP3A5* expressers, patients with the POR*28 allele had an approximately 20% lower TAC concentration-to-dose ratio than those with POR*1/*1. However, the POR*28 polymorphism had no effect on the TAC concentration/dose ratio in *CYP3A5* non-expressers. This can be explained by the role of POR, which provides electrons and enhances CYP activity.

Ethnicity may affect the expression of *POR* and thereby TAC metabolism. As the minor allele frequency of POR*28 was 20.0% in African Americans, 28.6% in Caucasians, and 38.9% in Asians [41], Asians are thought to be more affected by POR*28. Unfortunately, we could not compare the POR*28 effects by ethnicity, due to the small number of studies in non-Asian populations. Further studies are needed.

Our findings should be interpreted considering the following limitations. First, only six retrospective studies were included. Second, some heterogeneity existed, possibly due to the difference in the analytic methods used to determine concentrations and target concentrations. Last, although we used standardized trough concentrations after considering weight and dose, we could not adjust several factors (e.g., concurrent drugs), which can affect TAC concentrations, due to the lack of individual data. 

Nevertheless, our meta-analysis demonstrated that the POR*28 polymorphism affects the TAC standardized trough concentration during the early post-transplantation period in adult renal transplant recipients, especially *CYP3A5* expressers. *POR* and *CYP3A5* genotyping might help to adjust appropriate TAC doses to reach target trough concentrations, leading to better treatment outcomes.

## Figures and Tables

**Table 1:** Search strategy.

No	Search Term	PubMed	Web of Science	Embase
#1	(tacrolimus) OR (FK506) OR (FK-506) OR (calcineurin inhibitor) OR (Prograf) OR (immunosuppress*)	481,508	153,419	391,339
#2	(kidney transplant*) OR (kidney graft*) OR (kidney allograft*) OR (renal transplant*) OR (renal graft*) OR (renal allograft*)	190,248	202,994	324,655
#3	#1 and #2	48,448	31,892	78,405
#4	(POR) OR (P450 oxidoreductase) OR (cytochrome P450 oxidoreductase) OR (CYPOR)	138,663	16,910	70,583
#5	(polymorph*) OR (variant*) OR (mutation*) OR (genotyp*) OR (phenotyp*) OR (haplotyp*) OR (SNP) OR (rs1057868) OR (Ala503Val) OR (A503V)	2,146,909	2,144,583	2,821,213
#6	#4 and #5	25,468	1794	3279
#7	#3 and #6	460	53	73

**Table 2:** The characteristics of included studies.

First Author, Year	Ethnic Background	N (Male %)	Age, Year (SD)	Weight, kg (SD)	POR*28 Allele Frequency (%)	Post-Transplantation Day	Initial Dose	Target Trough Level, ng/mL	Coadministration	Genotyping Methods	Quantification Methods	NOS
Elens et al., 2014 [26]	Caucasian, Asian, Africa-American, Others	127 * (60.2)	49.5 (15.3)	72.6 (16.6)	22.1	10	NA	5~15	MMF or azathioprine, corticosteroids	TaqMan assay	MEIA	9
Kurzawski et al., 2014 [27]	Caucasian	241 (55.6)	45.8 (12.4)	73.2 (13.9)	26.4	7	100 ng/kg/day	10~15	MMF, corticosteroids	TaqMan assay	CMIA	9
Li et al., 2014 [28]	Asian	240 (67.1)	41.0 (12.2)	57.9 (10.1)	35.6	6~8	100 ng/kg, bid	9~14	MMF, steroids	SNaPshot assay	MEIA	7
Zhang et al., 2015 [29]	Asian	83 (72.3)	40.4 (11.3)	62.0 (9.4)	39.8	7	NA	10~15	MMF, steroids	PCR-RFLP	Emit 2000 Tacrolimus assay	9
Liu et al., 2016 [30]	Asian	154 (NA)	40.0 (10.9)	59.8 (10.7)	34.1	7	50~75 ng/kg, bid	5~8	MMF, prednisolone	PCR-RFLP	MEIA	8
Phupradit et al., 2018 [31]	Asian	216 (61.1)	43.0 (14.6)	57.1 (11.3)	32.4	7	100 ng/kg/day	4~8	Mycophenolic acid, corticosteroids or basiliximab	TaqMan assay	CMIA	9

bid: twice a day; CMIA: chemiluminescent microparticle immunoassay; MEIA: microparticle enzyme immunoassay; MMF: mycophenolate mofetil; NA: not available; NOS: Newcastle–Ottawa score; PCR–RFLP: polymerase chain reaction–restriction fragment length polymorphism; SD: standard deviation. * Of the total population of 184, only 127 blood samples were obtained on day 10.

**Table 3:** A sensitivity analysis of the association between POR*28 carriers and standardized trough concentration (ng/mL per mg/kg) of tacrolimus by sequentially excluding each study.

Excluded Study	Heterogeneity I^2^ (%)	Statistical Model	Mean Difference [95% CI]
None	55	Random	−11.67 [−14.16, −9.19]
Elens et al., 2014 [26]	62	Random	−8.68 [−15.95, −1.42]
Kurzawski et al., 2014 [27]	53	Random	−9.51 [−16.32, −2.70]
Li et al., 2014 [28]	52	Random	−9.61 [−16.04, −3.17]
Zhang et al., 2015 [29]	54	Random	−6.97 [−13.17, −0.76]
Liu et al., 2016 [30]	29	Fixed	−5.38 [−11.17, 0.40]
Phupradit et al., 2018 [31]	58	Random	−8.84 [−16.59, −1.09]

CI: confidence interval.

## Data Availability

Not applicable.
